# Generalized statistical mechanics of cosmic rays: Application to positron-electron spectral indices

**DOI:** 10.1038/s41598-018-20036-6

**Published:** 2018-01-29

**Authors:** G. Cigdem Yalcin, Christian Beck

**Affiliations:** 1 Department of Physics, Istanbul University, 34134 Vezneciler, Istanbul Turkey; 2 School of Mathematical Sciences, Queen Mary University of London, Mile End Road, London, E1 4NS UK

## Abstract

Cosmic ray energy spectra exhibit power law distributions over many orders of magnitude that are very well described by the predictions of *q*-generalized statistical mechanics, based on a *q*-generalized Hagedorn theory for transverse momentum spectra and hard QCD scattering processes. QCD at largest center of mass energies predicts the entropic index to be $q=\frac{{\bf{13}}}{{\bf{11}}}$. Here we show that the escort duality of the nonextensive thermodynamic formalism predicts an energy split of effective temperature given by Δ ${\boldsymbol{k}}{\boldsymbol{T}}{\boldsymbol{=}}{\boldsymbol{\pm }}\frac{{\bf{1}}}{{\bf{10}}}{\boldsymbol{k}}{{\boldsymbol{T}}}_{{\boldsymbol{H}}}{\boldsymbol{\approx }}{\boldsymbol{\pm }}{\bf{18}}$ MeV, where *T*_*H*_ is the Hagedorn temperature. We carefully analyse the measured data of the AMS-02 collaboration and provide evidence that the predicted temperature split is indeed observed, leading to a different energy dependence of th*e e*^+^ and *e*^−^ spectral indices. We also observe a distinguished energy scale E^*^ ≈ 50 GeV where th*e e*^+^ and *e*^−^ spectral indices differ the most. Linear combinations of the escort and non-escort *q*-generalized canonical distributions yield excellent agreement with the measured AMS-02 data in the entire energy range.

## Introduction

Statistical mechanics is a universal formalism based on the maximization of the Boltzmann-Gibbs-Shannon entropy subject to suitable constraints. Despite its universal validity and success for short-range equilibrium systems, the applicability of the Boltzmann-Gibbs formalism has severe restrictions: It is not valid for nonequilibrium systems, it is not valid for systems with long-range interactions (such as gravity), and it is not valid for systems with a very small volume and fluctuating temperature (as probed in scattering processes of cosmic ray particles at very high energies). For these types of complex systems it is useful to generalize the formalism to a more general setting, based on the maximization of more general entropy measures which contain the Shannon entropy as a special case. Probably the most popular one of these generalizations is based on *q*-entropy (or Tsallis entropy), which leads to power law distributions (the so-called *q*-exponentials), but other generalized entropic approaches are possible as well^1–8^. In high energy physics, a recent success of the *q*-generalized approach is that excellent fits of measured transverse momentum spectra in high energy scattering experiments have been obtained^9^, based on an extension of Hagedorn’s theory^10–12^ to a *q*-generalized version and a generalized thermodynamic theory^13–19^. This includes recent experiments in the TeV region for *pp* and $p\bar{p}$ collisions^9,20–25^ but there is also early work on cosmic ray spectra^26,27^ and *e*^+^*e*^−^ annihilation^15,28,29^.

In this paper we systematically investigate the relevant degrees of freedom of the *q*-generalized statistical mechanics formalism at highest center of mass energies and develop an effective theory of energy spectra of cosmic rays, which are produced by scattering processes at extremely high energies (e.g. supernovae explosions). Some remarkable predictions come out of the formalism in its full generality. First of all, the parameter *q* is not an arbitrary fitting parameter but fixed at highest center of mass energies— by comparing the predicted power law decay for transverse momentum spectra with the hard QCD parton scattering amplitude^9^. In the parametrization we use in the current paper this leads to $\,q=\frac{13}{11}=1.1818$. Next, the average temperature parameter for QCD scattering processes and subsequent hadronisation is theoretically expected to be the Hagedorn temperature, it cannot be varied either but is fixed by theory. In fact, in this paper we will show that in full generality the *q*-generalized statistical mechanics predicts a small temperature splitting close to the Hagedorn temperature, by applying the escort and non-escort formalism of the theory^30,31^ (a manifestation of the so-called *q*-duality of the generalized statistical mechanics^4–7,32,33^). Remarkably, this temperature split prediction of the theory is confirmed by the recent AMS-02 measurements^34–38^ of fluxes for cosmic ray electrons and positrons, as we will show in this paper. The small temperature split leads to a different behavior of electron and positron spectral indices as a function of energy, which so far was not understood, but is here identified as a feature of the generalized statistical mechanics formalism.

## Background and previous work

Let us motivate the use of generalized statistical mechanics as an effective theory for cosmic rays in a bit more detail. Generally, it is well known that a large variety of different physical processes contribute to the observed spectrum of cosmic rays at the Earth. First of all, there is the primary statistics generated by the primary production and acceleration process of cosmic rays in sources such as supernovae, active galactic nuclei, heavy stars with stellar winds, or even the big bang itself. Not much is known about the primary production process, but of course QCD scattering processes at extremely high energies will play an important role. The primary spectrum is then modified by subsequent propagation through the interstellar medium, where magnetic turbulence, ionization, Coulomb interactions, Bremsstrahlung, inverse Compton and synchrotron processes play an important role, as well as fragmentation in the interstellar medium. There is not just one process but a whole spectrum of many different complex processes involved, which suggests the use of generalized statistical mechanics as an effective theory. Finally, when entering the heliosphere the cosmic ray flux is further modulated by solar activity^39,40^.

QCD scattering and self-similar hadronic fragmentation processes have been previously successfully described by *q* ≠ 1 theories^9,16–19^ and applied to data taken at LHC^23–25^. It is precisely these types of scattering processes that we postulate as main ingredients to imprint on the momentum spectrum of primary cosmic rays, an initial spectrum set by QCD that in its main features is basically conserved, while other important processes then only yield small perturbations of the initial spectrum set by QCD, at least in a statistical sense. Still subtle differences between electron and positron statistics are possible, as well as deviations from simple QCD behavior, which we will discuss in more detail in the following sections.

Among phenomenological approaches to understand high energy cascading scattering processes, power laws associated with Tsallis statistics are by now widely used by many groups^9,16–21^. In fact they yield surprisingly good fits of a variety of data sets for different systems. This approach uses the assumption that the highly excited and fragmented states formed in high energy collisions follow Tsallis statistics instead of Boltzmann statistics. Initially only regarded as a mathematical playground for more general versions of statistical mechanics, based on the maximization of more general entropy measures^1–4^ the approach has more recently led to more sophisticated theories which do produce excellent agreement with experimental data, much more beyond the original fitting approach. Recent models are now going much more in-depth on which specific type of formalism is appropriate, and relate the entropic index to QCD scattering processes in the perturbative regime^9,18^. But also the nonperturbative regime is accessible, where the generalized statistical mechanics formalism arises out of the self-similarity of the fragmentation process and can be related to the fractal structure of hadrons within the MIT bag model for hadron structure^16,17^. Moreover, Tsallis statistics is generally well-known to be highly relevant for velocity distributions in astrophysical plasmas (here these distributions come under the name *Kappa-distributions*, see, e.g.^41^ for a recent review). Importantly, an entropic index *q* ≠ 1 can also arise from nonequilibrium dynamics, in particular from suitable spatio-temporal fluctuations of an intensive parameter such as the local (inverse) temperature (the superstatistics approach^42^ as developed by Beck and Cohen (see note in the acknowledgements). In this paper, for the very first time, we apply these techniques borrowed from generalized statistical mechanics to analyse the AMS-02 data sets.

## Results

### *q*-dualities and theoretical prediction of a temperature split

While there are by now many different versions of the nonextensive formalism in high energy physics, each describing different aspects of different scattering systems, for cosmic rays consisting of electrons and positrons we start from one of the simplest versions: As in early work on cosmic ray data^26,27^ we start from *q*-generalized canonical distributions of the form

1 $$p(E)\sim \frac{{E}^{2}}{{(1+(q-\mathrm{1)}{\beta }_{0}E)}^{\frac{1}{q-1}}}.$$

Here *q* is the entropic index and *β*_0_ is an inverse temperature parameter. *E* is the energy of the particle. For *q* → 1 the ordinary Maxwell Boltzmann distribution

2 $$p(E)\sim {E}^{2}{e}^{-{\beta }_{0}E}$$

is recovered. The above distributions are *q*-generalized canonical distributions in the nonextensive formalism, *E*^2^ is a phase space factor. They maximize Tsallis entropy ${S}_{q}=\frac{1}{q-1}\sum _{i}\mathrm{(1}-{p}_{i}^{q})$ subject to suitable constraints^1–4,31^. They generate power law distributions for large energies *E* and are thus very well suited to fit power law distributions observed in cosmic ray physics. Defining the spectral index *γ* by $\gamma :=d\,\mathrm{log}\,p(E)/d\,\mathrm{log}\,E$ we obtain the result that a single distribution of the form Eq. (1) generates the spectral index

3 $$\begin{array}{cc}\gamma (E)=2-\frac{{\beta }_{0}E}{1+(q-1){\beta }_{0}E}\to 2-\frac{1}{q-1} & ({\beta }_{0}E\to {\rm{\infty }})\end{array}.$$

A physical motivation for the occurrence of the above asymptotic power-law distributions can be given by the superstatistics approach, which quite generally describes the effect of big temperature fluctuations in nonequilibrium situations^42^. We can write Eq. (1) in the equivalent form

4 $${\int }_{0}^{\infty }d\beta f(\beta ){E}^{2}{e}^{-\beta E}=\frac{{E}^{2}}{{\mathrm{(1}+(q-\mathrm{1)}{\beta }_{0}E)}^{\mathrm{1/(}q-\mathrm{1)}}}$$

where *f*(*β*) is a *χ*^2^ distribution with *N* = 2/(*q* − 1) degrees of freedom and ${\beta }_{0}={\int }_{0}^{\infty }d\beta \,\beta f(\beta )=\langle \beta \rangle$ is the average of a fluctuating random variable *β* that is distributed with *f*(*β*). One can easily check that $q=\langle {\beta }^{2}\rangle /{\beta }_{0}^{2}$, so *q* − 1 is a measure of the width of the inverse temperature fluctuations^43,44^. The physical interpretation is that power law Boltzmann factors (1 + (*q* − 1)*β*_0_*E*)^−1/(*q*−1)^ arise from ordinary Boltzmann factors *e*^−*βE*^ in nonequilibrium situations where there is a distribution *f*(*β*) of inverse temperatures *β*, after integrating over all possible *β* weighted with *f*(*β*). The relevance of temperature fluctuations in cosmic ray physics has been previously emphasized in^27,29^.

Our main goal in the following is to derive physically plausible values for *q* and *β*_0_ for cosmic ray energy spectra, in particular for *e*^+^ and *e*^−^ cosmic rays, and to proceed to linear combinations of distributions of type (1). To this end we assume that the underlying primary production process of the cosmic rays is QCD parton scattering at largest possible energies. Essentially protons (which make up the dominant component of cosmic rays) collide at extremely high energies and produce other particles in the process. In these high energy scattering processes and subsequent hadronisation cascades a large number of baryons and anti-baryons as well as mesons are produced. These hadrons ultimately decay to stable particles, including electrons and positrons, which continue their way as cosmic rays, but imprinted on their statistical transverse momentum distribution is the original high-energy scattering and hadronisation process that took place at the Hagedorn temperature.

Wong *et al*.^9^ have derived that for hard parton QCD scattering processes the asymptotic power law dependence of the cross section imposed by the leading QCD scattering amplitude is $E\frac{{d}^{3}\sigma }{d{p}^{3}} \sim {E}^{-\frac{9}{2}}$ (see their Eqs (45) and (46) in^9^) which in our parametrization using the phase space factor *E*^2^ implies $p(E) \sim {E}^{-\frac{7}{2}}$. Thus QCD at largest energies fixes in our formula (1) the parameter *q* to be

5 $$2-\frac{1}{q-1}=-\frac{7}{2}\,\Longleftrightarrow \,q=\frac{13}{11}=\mathrm{1.1818.}$$

For QCD scattering processes, the average temperature 1/*β*_0_ should be essentially given by the Hagedorn temperature. To get the precise relation, we recall that in the nonextensive formalism there is a second important canonical distribution, the so-called escort distribution^30,31^. This is given by

6 $$\hat{p}(E)\sim {E}^{2}\frac{1}{{\mathrm{(1}+(\hat{q}-\mathrm{1)}{\hat{\beta }}_{0}E)}^{\frac{\hat{q}}{\hat{q}-1}}}$$

where $\hat{q}$ and ${\hat{\beta }}_{0}$ are the entropic index and inverse temperature parameter for the escort distribution. Basically, the escort distribution is obtained by raising all given microstate probabilities *p*_*i*_ to the power *q* and renormalizing this distribution (see^30,31^ for details). In this way, for any *q* ≠ 1 two degrees of freedoms arise out of the generalized statistical mechanics treatment: Escort distributions and non-escort distributions. Both formalisms can be mapped onto each other, this is called the *escort duality*. The escort duality can also be understood in terms of superstatistics, a related concept was called type-A and type-B superstatistics in^42^. There are actually two *q*-dualities in the nonextensive formalism, corresponding to the replacements *q* → 2 − *q* and *q* → 1/*q*. These are sometimes called *additive* and *multiplicative* duality^4,33^ and they can be combined to give the *escort* duality $q\to 2-\frac{1}{q}$.

In our case, QCD fixes the power law index

7 $$n:=\frac{1}{q-1}=\frac{\hat{q}}{\hat{q}-1}$$

to $n=\frac{11}{2}$ at largest energies. From this we derive a relation between *q* and $\hat{q}$:

8 $$q=2-\frac{1}{\hat{q}}\iff \hat{q}=\frac{1}{2-q}$$

which is just the escort duality. The distributions *p*(*E*) given in Eq. (1) and $\hat{p}(E)$ given in Eq. (6) describe the same physics and should be the same, no matter whether we mathematically use the escort or non-escort formalism. This yields a relation between the inverse temperature parameters *β*_0_ and ${\hat{\beta }}_{0}$:

9 $$(q-\mathrm{1)}{\beta }_{0}=(\hat{q}-\mathrm{1)}{\hat{\beta }}_{0},$$

which can be solved to give ${\hat{\beta }}_{0}=\frac{q-1}{\hat{q}-1}{\beta }_{0}=\mathrm{(2}-q){\beta }_{0}$. In terms of average temperature $kT={\beta }_{0}^{-1}$ the fixed index *n* implies the existence of two different temperature parameters:

10 $$\hat{T}=\frac{1}{2-q}T$$

If we define the Hagedorn temperature *T*_*H*_ as the average of these two degrees of freedom,

11 $${T}_{H}=\frac{1}{2}(\hat{T}+T)$$

we obtain ${T}_{H}=\frac{1}{2}(\frac{1}{2-q}+1)T=\frac{1}{2}\frac{3-q}{2-q}T$. It then follows $T=2\frac{2-q}{3-q}{T}_{H}$, and $\hat{T}=\frac{2}{3-q}{T}_{H}$ so that ${\rm{\Delta }}T:=\hat{T}-$ ${T}_{H}=\frac{q-1}{3-q}{T}_{H}$. In particular, for the QCD hard scattering processes with $n=\frac{11}{2}$ we have $q=\frac{13}{11}=1.1818$, $\hat{q}=\frac{11}{9}=1.2222$ and the predicted split in temperature is given by

12 $$T=\frac{9}{10}{T}_{H}={T}_{H}-\frac{1}{10}{T}_{H},$$

13 $$\hat{T}=\frac{11}{10}{T}_{H}={T}_{H}+\frac{1}{10}{T}_{H}\mathrm{.}$$

This means that two different effective temperatures degrees of freedom come out of the formalism, and this is an experimentally testable prediction that one can check on the measured cosmic ray data. There are slight uncertainties in the knowledge of the precise value of the Hagedorn temperature in the literature, for our fits in the following we use the value *kT*_*H*_ = 180 MeV as in^12^ getting *kT* = 162 MeV and $k\hat{T}$ = 198 MeV.

The main idea of our derivation of the above temperature split is that in the *q*-generalized formalism the physical meaning is not directly connected with the entropic index *q* but with the power law exponent *n*, since the latter one is directly measurable. However, it does not matter if one writes *n* in either the form *n* = 1/(*q* − 1) or *n* = *q*/(*q* − 1). But if one does both then the temperature must be different in each of these cases, as shown above. This temperature splitting occurs only for *q* ≠ 1, whereas ordinary statistical mechanics with *q* = 1 has a temperature fixed point.

### Comparison with AMS-02 measurements

**Figure 1:**
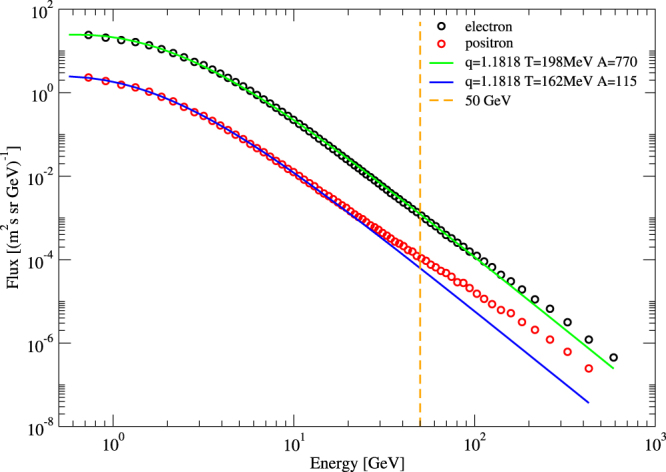
Flux Φ(*E*) of *e*^+^ and *e*^−^ primary cosmic ray particles of energy *E* as measured by AMS-02^34–38^ and theoretical prediction of the *q*-generalized Hagedorn theory (solid lines).

Let us now compare our theoretical predictions with the measurements of the AMS-02 collaboration^34–38^. Figure 1 shows that formula (1) with the predicted *q*-value $q=\frac{13}{11}$ and the two predicted temperatures $T={T}_{H}\pm \frac{1}{10}{T}_{H}$ yields an excellent fit of the AMS data up to energies of about 50 GeV. Remarkably, electrons are described by $\hat{T}=\frac{11}{10}{T}_{H}$ and positrons by $T=\frac{9}{10}{T}_{H}$, thus giving physical meaning to the two degrees of freedom that come out of the generalized statistical mechanics theory. The fits are very sensitive to the *q*-values used, up to 3–4 digits of precision in *q* can be distinguished, confirming the QCD value *q* = 1.1818 derived from theory, as well as the predicted temperature split.

The error bars of the data that are plotted in Fig. 1 (as listed by AMS-02) are small, when these error bars are plotted in our logarithmic plot, they are of the same order of magnitude as the size of the symbols, and have therefore been omitted in our Fig. 1 for reasons of better visibility. Note that in Fig. 1 all parameters used are predicted, and not fitted. Excellent agreement is obtained up to energies of about 50 GeV. The only fitting parameters used in Fig. 1 is the absolute strength of the flux (corresponding to the vertical position of the curves in the logarithmic plots): The electron flux amplitude *A*_−_ = 770 is about 7 times stronger than the positron amplitude *A*_+_ = 115.

Our *q*-generalized statistical mechanics has no tool to predict the amplitude parameters *A*_±_ from first principles, it can only give predictions on the shape of the spectrum, given some fixed amplitude parameter for a given particle species. The conventional wisdom to explain why *A*_−_ ≫*A*_+_ is that electrons are mainly produced in the primary acceleration process, whereas positrons are believed to be mainly secondary particles arising from collisions with the interstellar plasma. An alternative approach, more in line with what we see from the actual fits of the *e*^+^ and *e*^−^ spectra, would be that both positrons and electrons are initially produced in the same primary QCD cascading process with similar amplitude (and with the same *q*-value and only a slightly different Hagedorn temperature), but that then an energy-independent absorption process sets in where anti-particles (positrons) have a higher probability of being absorbed than particles (electrons), leading finally to the observed ratio *A*_+_/*A*_−_ ≈ 1/7. One might speculate that this absorption process has to do with the (unknown) process that leads to CP symmetry breaking in the universe and the dominance of matter over anti-matter abundance. As said, a full understanding of the amplitude ratio *A*_+_/*A*_−_ is out of reach of our simple statistical mechanics model, but has been the subject of other papers.

Another important feature to discuss, important for the low-energy end of the spectrum, are solar modulation effects. At low energies (typically *E* ≤ 1 GeV) the flux of *e*^+^ and *e*^−^ varies in time due to the changing pattern of solar activity, thus making the measured flux of electron and positron cosmic rays time dependent. Solar modulations occur because of interactions of cosmic rays entering the solar heliosphere; they are due to charge sign dependent propagation in the solar magnetic field. For a review, see^39^. It is clear that our prediction, based on a simple statistical mechanics model which does not know about this effect, cannot give quantitative predictions related to the solar modulation effects. What is measured in the experimental data is the time-integrated effect, but this effect is not zero but always depletes the spectrum at very low energies. What our analysis and the good agreement with the data seems to indicate is that the averaged effect of solar modulation is negligible at energies down to around 1 GeV, at least in the logarithmic scale that is being used in Fig. 1.

Let us use previous work to estimate the order of magnitude of the expected solar periodic modulation. Fig. 20 of^40^ (based on PAMELA data) indicates that at an energy of 0.7 GeV, the lowest energy considered in our Fig. 1, the half-yearly measured flow changes by a factor of up to 1.5, due to the solar modulation effect. Although the effect is highly significant for a detailed understanding of the time dependence of the low-energy spectra, a factor of 1.5 is hardly visible in the logarithmic plot of our Fig. 1, where it just leads to a shift of the data that has the same order of magnitude as the symbol size, given the logarithmic scale of the figure. That said, an interesting future project would be a precision comparison of the time-dependent fitting parameters of the generalized statistical mechanics approach with time-dependent data as presented e.g. in^40^.

**Figure 2:**
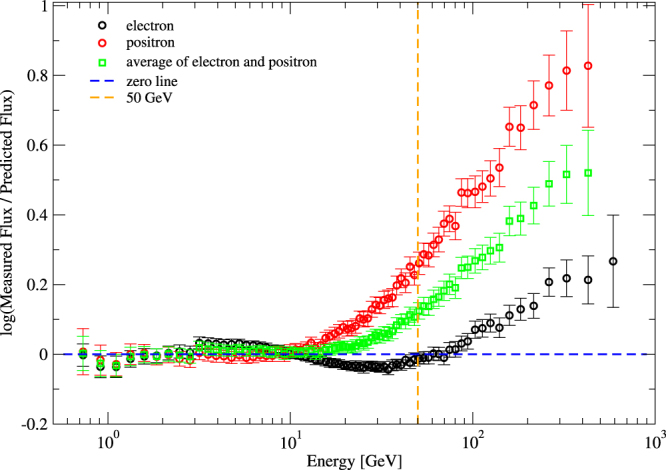
Ratio of measured flux to predicted flux as given by Eq. (1) as a function of energy *E* of the cosmic ray particles. The data correspond to AMS-02 measurements of electrons, positrons, and both species together. The vertical line indicates the energy *E* = 50 GeV.

Let us now proceed to higher energies. In Fig. 2 it is shown that at an energy of the order 50 GeV our formula based on a single *q*-exponential as given in Eq. (1) starts to deviate significantly from the measured flux data. Positrons start to deviate earlier than electrons. We will later give a suitable definition of a joint transition point by analysing the ratio of local positron and electron spectral indices, which is an amplitude-independent quantity.

**Figure 3:**
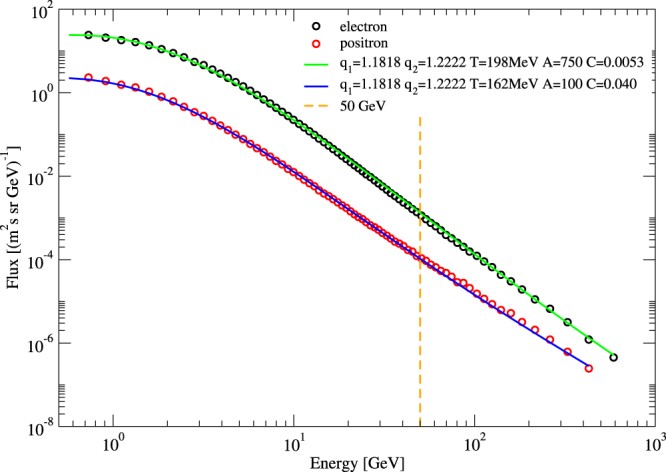
The measured AMS-02 data are very well fitted by linear combination of escort and non-escort distributions (solid lines).

Apparently, an additional process starts to contribute to the cosmic ray flux for energies *E* larger than about 50 GeV. We found that this crossover is very well described by a linear combination of generalized canonical distributions where the entropic index takes on two values, namely the QCD value 13/11 = 1.1818 and the escort value 11/9 = 1.2222, evaluated at temperature *T* for positrons and $\hat{T}$ for electrons. Figure 3 shows that in the entire energy range the measured cosmic ray flux is very well fitted by the linear combination

14 $${P}_{\pm }(E)={A}_{\pm }(\frac{{E}^{2}}{{\mathrm{(1}+(q-\mathrm{1)}{\beta }_{0}E)}^{\frac{1}{q-1}}}+{C}_{\pm }\frac{{E}^{2}}{{\mathrm{(1}+(\hat{q}-\mathrm{1)}{\beta }_{0}E)}^{\frac{1}{\hat{q}-1}}})$$

where *C*_+_ ≈ 0.04 for positrons, *C*_−_ ≈  0.0053 for electrons, and *β*_0_ = *T*^−1^, respectively ${\hat{T}}^{-1}$.

Apparently, the power law exponent $-\frac{1}{\hat{q}-1}=-4.5$ is larger than the power law exponent $-\frac{1}{q-1}=-5.5$, and hence the former term dominates the large energy behavior if *E* → ∞. The crossover scale observed in the AMS data is roughly 50 GeV. We may physically interpret this crossover again as an effect of the two degrees of freedom (escort and non-escort) that are predicted by the *q*-generalized statistical mechanics formalism: The physically relevant generalized canonical distribution is a mixed state of both, and hence at smaller energies the behavior is dominated by the power law exponent $\frac{1}{q-1}$, whereas at higher energies it is dominated by $\frac{1}{\hat{q}-1}$.

Let us summarize the main assumptions underlying our approach. The generalized statistical mechanics presented in this paper gives concrete predictions on the shape of the flux spectrum as a function of observed energy of the particles, but it relies on the assumption that mainly QCD scattering and fragmentation process determine the shape of the spectrum, whereas other processes are either neglected or absorbed in terms of an effective description. While we do predict general spectral shapes based on generalized statistical mechanics methods, we certainly cannot give predictions on the size of the absolute flow of particles as modified by the local astrophysical environment. In particular, very low energy spectra of electrons in the MeV region (such as those discussed in^45^) are certainly out of reach of the current formalism.

The generalized statistical mechanics model applies to both primary and secondary production processes of electrons and positrons, just the relevant parameter *q* is different, depending on the center of mass energy *E*_*CMS*_ considered. In particle physics scattering processes at typical LHC center of mass energies *E*_*CMS*_ in the TeV region, *q* is observed to be typically around *q* ≈ 1.10^9,18,46^ and one has theoretical predictions that for *E*_*CMS*_ → ∞, *q* should approach 1.22^15^. The latter value fits much better the observed cosmic ray spectra, hence our conjecture is that both electron and positron spectra get their *q* ≈ 1.2 imprinted already in the primary production process, at significantly higher energies than the TeV region. The secondary scattering processes then have a *q* more in the region like the LHC data, *q* ≈ 1.1, so this secondary power law decays stronger and can thus be neglected as compared to the primary power spectrum which decays slower since *q* is bigger. Generally, the power laws decay as ${E}^{2-\frac{1}{q-1}}$, so the largest *q* dominates the relevant spectrum. In this context it is interesting to note that at very large center of mass energies the existence of both a limiting *q* and a limiting *β* has been predicted on the basis of thermodynamic consistency relations^47,48^. A systematic comparison with LHC scattering data for various particle species was presented in^46^.

### WIMP annihilation and other new physics

An observed crossover feature in the cosmic ray flux pattern of particles and anti-particles such as the one in Fig. 3 can have many origins, ranging from conventional astrophysical explanations to more speculative explanations such as dark matter annihilations and/or decay. Let us here concentrate on a possible interpretation in terms of WIMP physics, following the ideas presented in^49–55^. If there is a weakly interacting massive particle (WIMP) underlying dark matter, then WIMP annihilation and/or WIMP decay can modify the relative abundance of *e*^+^ and *e*^−^ flux. In particular, a distinct change of behavior of the flux is expected at a threshold energy given by the WIMP mass. Presently WIMP masses of ~50 GeV near the *Z*^°^ pole are still consistent with particle collider experiments in a variety of models and have been proposed as an explanation for the observed *γ*-ray excess from the center of the galaxy^49–53^. Also, a recent analysis of the AMS-02 antiproton flux data appears to be consistent with a WIMP mass of about 50 GeV^54,55^. Interestingly, our fit of the electron positron data seems to indicate *A*_−_*C*_−_ = *A*_+_*C*_+_, meaning the excess flux contribution starting to dominate in Eq. (14) from 50 GeV onwards has the same amplitude for electrons and positrons.

**Figure 4:**
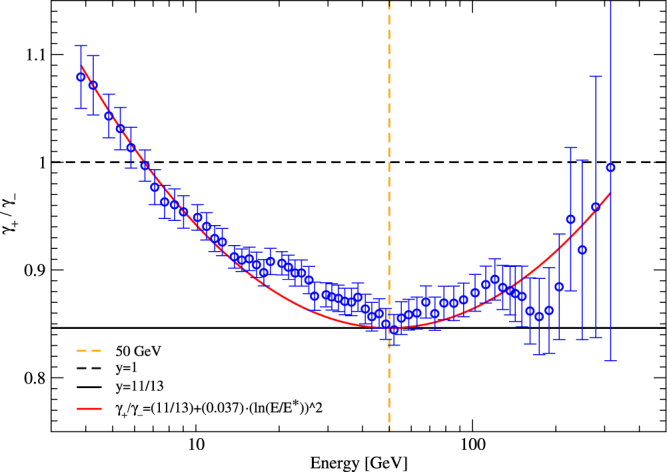
Ratio $\frac{{\gamma }_{+}}{{\gamma }_{-}}$ of positron and electron spectral indices as a function of energy *E* (data from the AMS-02 collaboration^38^). T he curve $\frac{{\gamma }_{+}}{{\gamma }_{-}}$ has a minimum at *E* = E^*^ = (50 ± 10) GeV and is well fitted by Eq. (15).

To better characterize the crossover point, we define it as the point where the spectral indices *γ*_+_ and *γ*_−_ of positrons and electrons differ the most: |*γ*_+_ − *γ*_−_| = *max* implies that *γ*_+_/*γ*_−_ has a minimum. Figure 4 shows the ratio *γ*_+_(*E*)/*γ*_−_(*E*) as a function of energy *E* as measured by AMS-02^38^ (the error bars of the ratio were estimated by standard methods). We observe that there is a local minimum at *E* = E^*^ = (50 ± 10) GeV, at this special point we have *γ*_+_ − *γ*_−_ = $\frac{1}{2}$ and *γ*_+_/*γ*_−_ = 0.8462 = 11/13 = *q*^−1^ (Fig. 3 in^38^ indicates *γ*_+_ = −2.75 and *γ*_−_ = −3.25). It is remarkable that the unknown crossover process taking place at ${E}^{\ast }$ satisfies ${\gamma }_{+}({E}^{\ast })=\hat{\gamma }-\frac{1}{4}$ and ${\gamma }_{-}({E}^{\ast })=\gamma +\frac{1}{4}$, so there is an antisymmetric correction $\pm \frac{1}{4}$ to the scaling exponents $\gamma =2-\frac{1}{q-1}$ and $\hat{\gamma }=2-\frac{1}{\hat{q}-1}$ associated with $q=\mathrm{13/11}$ and $\hat{q}=\mathrm{11/9}$. Figure 4 indicates that over a wide range of energies centered around E^*^ = 50 GeV the ratio *γ*_+_/*γ*_−_ exhibits a quadratic logarithmic energy dependence,

15 $$\frac{{\gamma }_{+}}{{\gamma }_{-}}(E)={q}^{-1}+C{(\mathrm{ln}\frac{E}{{E}^{\ast }})}^{2}$$

where *q* = 13/11, *C* = 0.037. The minimum at E^*^ corresponds to a distinguished superstatistical state satisfying *N*_−_ = *N*_+_ + 1, i.e. the *χ*^2^-distributions of inverse temperatures that are relevant for electrons and positrons differ by precisely 1 degree of freedom at this point. Physically this could be interpreted in terms of an additional particle degree of freedom that does influence the temperature fluctuations and is seen by electrons, but not by positrons.

Note that QCD on its own (without any *q*-generalized statistical mechanics) predicts the same spectral indices *γ*_+_ = *γ*_−_ for positrons and electrons. But the AMS-02 data (and previous data from PAMELA^56^) have shown that there is a significant difference between spectral indices of electrons and positrons, an asymmetry that is not fully understood so far. Whatever the theoretical explanation, the measured difference |*γ*_+_ − *γ*_−_|(*E*) as a function of energy can be regarded as a measure of the significance of the new physics underlying this difference. We see from the data analysed in Fig. 4 that the deviation is strongest at *E* = E^*^ ≈ 50 GeV. This observation is based on the measured AMS-02 data and is independent of any model assumption. It is intriguing to notice that the deviation is strongest at a mass scale that is the same as the extracted WIMP mass for other data sets such as the *γ* ray flux from the center of the galaxy and the antiproton flux data^50–52,54,55^.

Summarizing, our analysis of the very precise AMS-02 data has shown that for cosmic rays with an energy below the order of magnitude 50 GeV the observed statistics of both electrons and positrons is well described by what one would expect from QCD at largest possible center of mass energies *E*_*CMS*_ → ∞, a *q*-statistics with *q* = 13/11 (equivalent to a power law decay with exponent −5.5), and a temperature given by the Hagedorn temperature but with a small split in temperature, as expected from the *q*-generalized formalism in its full generality. While there are certainly important further processes for electrons and positrons on their way to Earth, such as e.g. energy loss by inverse Compton and synchrotron processes, our data analysis suggests that these additional processes do not significantly modify the primary entropic index *q* set by QCD at largest center of mass scattering energies. Indeed generalized statistical mechanics with *q* ≠ 1 has been shown in many previous papers to be relevant for QCD and hadronisation cascades, but less so for electromagnetic interaction processes which are better embedded into ordinary *q* = 1 statistical mechanics. So our main hypothesis, supported by the data, is that the primary entropic index *q* ≠ 1 set by QCD is essentially conserved, at least in a statistical sense, even if many further electrodynamical processes accompany the individual electrons and positrons on their way to Earth. But QCD alone cannot explain the spectra measured by AMS-02 in the high-energy region: What we actually observe is a transition point around E^*^ ≈ 50 GeV where the observed power law decay exponent switches from −5.5 to −4.5, and where one needs an additional mechanism to explain the excess of electrons and positrons as compared to the original QCD prediction. As WIMPs can decay into *e*^+^*e*^−^ it is natural to associate this observed increased flux to some new physics associated with WIMP decay. However, very recent experiments seem to provide growing evidence that WIMPs might not exist at all^57,58^. In this case a more conservative interpretation of the transition would be that both escort and non-escort distributions are realized in QCD scattering processes, with a crossover scale E^*^ where the escort index −4.5 starts to dominate the behavior.

## Discussion

In this paper we have applied *q*-generalized statistical mechanics methods to high energy scattering processes, which lie at the root of the production process of cosmic rays. These QCD scattering processes are thermodynamically described by the Hagedorn temperature, they are very similar in their momentum characteristics to the collision processes of TeV protons that are performed in experiments on the Earth^9,22^ except that they can take place at much higher center of mass energy. Stable particles such as electrons and positrons finally arise out of the hadronisation cascade by decays of pions, neutrons and other hadrons, and they memorize the momentum statistics of the Hagedorn fire ball. In this paper we carefully investigated the discrete degrees of freedom that are contained in the *q*-generalized statistical mechanics describing this. We showed that there are basically two degrees of freedom in the formalism that correspond to escort and non-escort distributions, and which allowed us to identify different statistical behavior of particle and anti-particle degrees of freedom, as observed in the measured cosmic ray spectra.

When comparing with the experimental data, the result of our analysis were two distinct energy scales: A large energy scale of about (50 ± 10) GeV, where the spectral indices of *e*^+^ and *e*^−^ differ the most, and a small energy scale of about (18 ± 1) MeV, which corresponds to a splitting of the effective Hagedorn temperature. The former energy scale sets the scale where the escort distribution power law starts to dominate the non-escort power law. The latter energy leads to a slightly different temperature statistics for electrons and positrons.

An interesting observation is that the theoretically predicted and experimentally observed split in Hagedorn temperature of $\frac{q-1}{3-1}k{T}_{H}\approx \mathrm{(18}\pm \mathrm{1)}$ MeV coincides with the mass scale of the recently postulated protophobic gauge boson^59–63^. A physical interpretation could be that some of the kinetic energy represented by the Hagedorn temperature is either absorbed or enhanced by this exotic postulated protophobic gauge boson should it exist. This would potentially be a quantum manifestation of the observed temperature split. By definition a protophobic gauge boson as introduced in^59^ couples to neutrons but not to protons. Assuming that electrons in cosmic rays arise to a significant part from *β*-decays of neutrons, then this could influence the observed momentum statistics and effectively lead to a slight asymmetry in the temperature statistics of electrons (which arise from neutron decays) as compared to that of positrons (which don’t). While the existence of the protophobic gauge boson still requires independent experimental verification, it is interesting that the *q*-generalized Hagedorn theory allows for a possible embedding of these types of energy scales close to the Hagedorn temperature.

To conclude, we have shown that the different energy dependence of the spectral indices of positron and electron cosmic rays is well explained by a *q*-generalized Hagedorn theory. The value of the parameter *q* = 13/11 and the relevant temperature parameters were derived, they are not fitting parameters but theoretically determined and appear to be in excellent agreement with the measurements of the AMS-02 collaboration for *E* < E^*^. The generalized statistical mechanics formalism together with the AMS-02 data analysed indicates the existence of two energy scales that could be potentially associated with new physics, a low-energy temperature split in Hagedorn temperature given by $\frac{1}{10}k{T}_{H}\simeq 18$ MeV and a crossover scale ${E}^{\ast }\simeq 50$ GeV where a process beyond QCD sets in, of unknown nature. T hese two energy scales appear to coincide with the mass scale of the recently postulated protophobic gauge boson^59–63^ and the mass scale of a WIMP that could explain the excess of *γ* rays from the galactic center^49–53^.
